# Adaptation to statins restricts human tumour growth in *Nude *mice

**DOI:** 10.1186/1471-2407-11-491

**Published:** 2011-11-22

**Authors:** Julie Follet, Lionel Rémy, Vincent Hesry, Brigitte Simon, Danièle Gillet, Pierrick Auvray, Laurent Corcos, Catherine Le Jossic-Corcos

**Affiliations:** 1 INSERM U613-ECLA and IFR148-ScInBioS, Université Européenne de Bretagne, Université de Bretagne Occidentale, Faculté de médecine, 22 avenue Camille Desmoulins, 29200 Brest, France; 2 INSERM U865, Faculté de Médecine RTH Laennec, 7 rue Guillaume Paradin, 69372 Lyon cedex 08, France; 3 C.Ris Pharma, Parc Technopolitain - Atalante Saint-Malo, 35400 Saint Malo, France

**Keywords:** Statins, Gastric cancer, *Nude *mice, Apoptosis, Angiogenesis

## Abstract

**Background:**

Statins have long been used as anti-hypercholesterolemia drugs, but numerous lines of evidence suggest that they may also bear anti-tumour potential. We have recently demonstrated that it was possible to isolate cancer cells adapted to growth in the continuous presence of lovastatin. These cells grew more slowly than the statin-sensitive cells of origin. In the present study, we compared the ability of both statin-sensitive and statin-resistant cells to give rise to tumours in *Nude *mice.

**Methods:**

HGT-1 human gastric cancer cells and L50 statin-resistant derivatives were injected subcutaneously into *Nude *mice and tumour growth was recorded. At the end of the experiment, tumours were recovered and marker proteins were analyzed by western blotting, RT-PCR and immunohistochemistry.

**Results:**

L50 tumours grew more slowly, showed a strong decrease in cyclin B1, over-expressed collagen IV, and had reduced laminin 332, VEGF and CD34 levels, which, collectively, may have restricted cell division, cell adhesion and neoangiogenesis.

**Conclusions:**

Taken together, these results showed that statin-resistant cells developed into smaller tumours than statin-sensitive cells. This may be reflective of the cancer restricting activity of statins in humans, as suggested from several retrospective studies with subjects undergoing statin therapy for several years.

## Background

Statins are widely used anti-hypercholesterolemia drugs and act through competitive inhibition of HMG-CoA reductase, the first enzyme controlling entry into the mevalonate pathway that leads, ultimately, to cholesterol and steroid hormone synthesis [1]. This drug family comprises both natural (lovastatin, simvastatin, pravastatin) and synthetic (fluvastatin, atorvastatin) molecules that efficiently lower LDL cholesterol levels [2]. Apart from their role in the control of cholesterol homeostasis, statins have been proposed to lower cancer incidence in several large trials, for colon, breast and lung cancers, among others [3,4]. However, other large trials have not confirmed this potential chemo-preventive effect [5]. The origin of the differences is not known, but a recent study indicated that these might partly depend on the particular allelic form of the HMGCR gene (encoding HMG-CoA reductase), as an alternative pre-mRNA splicing event - associated with a specific SNP genotype - leads to differences in the activity of the enzyme [6].

In addition to this cancer prevention potential, statins have long been known to trigger apoptosis in many cell culture models, to prevent or reduce tumour occurrence in animals or to reduce the incidence of metastases [7]. The tumour suppressive effect has been proposed to rely partly on the ability of statins to block production of farnesyl pyrophosphate or geranyl-geranyl pyrophosphate, which provide target proteins with C15 or C20 post-translational carbon chain additions that help signalling molecules like Ras or Rho anchor to the plasma membrane and drive cell proliferation [8,9]. Cholesterol deprivation might also hamper tumour cell proliferation by restricting the ability to renew membrane pools. Other effects, linked to reduced respiration potential, have also been proposed [10].

One question that arises is what would happen, in the apoptotic response, if the sensitivity of the cells to statins was reduced, either as an intrinsic or an acquired phenotype following long periods of drug intake? To address this question, we isolated a population of statin-resistant cells, which we named L50, that was stably resistant to a relatively high concentration of statins in culture (50 μM), derived from the human gastric cancer HGT-1 cell line [11]. These cells showed increased expression of pro-caspase-7, that we further showed to be under the positive control of SREBP-1 and SREBP-2 proteins, much like proteins from the mevalonate pathway and pro-caspase-2 [12]. *In vitro *growth of HGT-1 and L50 cells showed that the latter had a reduced growth rate, suggesting that, should intrinsic resistance to statins occur, it would not be associated with increased growth. The aim of the present study was to investigate cell growth parameters and marker gene expression in tumours developed in *Nude *mice from HGT-1 or L50 cells.

Our results showed that tumour growth was slower in L50 than in HGT-1 tumours, as found for cells grown *in vitro*. In addition, the over-expression of caspase-7 in L50 *vs *HGT-1 cells was maintained in tumours, further demonstrating phenotype stability. The lower growth of L50 tumours was associated with a strong reduction in cyclin B1. In addition, these L50 tumours showed over-expression of collagen IV, and reduced laminin 332, VEGF and CD34 levels, which may have restricted cell adhesion and neoangiogenesis. Taken together, these results suggest that adaptation to statins led to important phenotype modifications, which, collectively, conferred a reduced ability of tumour cells to grow in immuno-compromised mice.

## Methods

### Cell culture

HGT-1 human gastric cancer cells and HGT-1-derived L50 cells were grown at 37°C under a humidified atmosphere of 5% CO_2 _in DMEM (Dulbecco's modified Eagle's medium) (Lonza, Saint Beauzire, France), containing 4.5 g.L^-1 ^glucose and supplemented with 5% (v/v) foetal bovine serum without antibiotics (Gibco-Invitrogen, Cergy Pontoise, France) [11,13]. L50 cells had been selected following several weeks of permanent growth in presence of 50 μM lovastatin. Cell death no longer occurred after that period and phenotype stability was ascertained over several cell passages in the absence of lovastatin [11]. HGT-1 and L50 cells were grown and used for injection into *Nude *mice (see below).

### Tumour induction in *Nude *mice

Twenty healthy female Balb/c *Nude *mice (4 weeks-old) were obtained from Charles River (L'Arbresle, France). Animals were maintained for 7 days in a conventional animal care unit before the start of the study (INSERM U625, Rennes, France, Agreement No. 4016 from the French Ministries of Agriculture and Research). Animal experiments were performed according to ethical guidelines of animal experimentations.

Before cell injection, the mice were anesthetised by intra-peritoneal injection of pentobarbital (70 mg/kg; Sigma, France). The cells (2 × 10^7 ^cells/mouse in 200 μl of serum-free medium) were then inoculated by subcutaneous injection in the right flank of each mouse (10 mice per group). After cell inoculation, mice were observed for 2 h post-injection to ascertain that no health condition occurred. Tumour growth was followed during 31 days. The tumor volume was calculated according to the formula: (length × width^2^)/2 [14]. Randomization all along the covered period, including randomization to affect animals to the treatment groups, to pick up the mice from both groups and to measure the tumour volumes was used for all the experiment. At the end of this period, the mice were sacrificed and tumours were recovered for analysis.

### Protein extraction and western blotting analysis

Frozen tumours were rapidly homogenised in Phosphate Buffered Saline (PBS) and lysed in ripa buffer (50 mM Tris HCl pH7.4, 150 mM NaCl, 0.5% (wt/v) Sodium deoxycholate, 0.1% SDS, 1% NP40, 1 mM EDTA, 1 mM PMSF) containing protease inhibitor cocktail (Roche, Meylan, France) and phosphatase inhibitor (Active motif) for 10 min at 4°C. Sixty micrograms of proteins were boiled in Laemmli buffer for 5 min, separated by SDS-PAGE using 12% or 15% polyacrylamide gels and blotted onto polyvinyl difluoride membranes (GE Healthcare). Non specific binding sites were blocked for 1 h at room temperature by 5% (wt/v) fat-free milk before an overnight incubation at 4°C with specific rabbit anti-human antibodies: procaspase-3, -6, -7 or -9, aurora kinase A and B, Bcl-2, Bax and survivin (Cell Signalling Technology-Ozyme, Saint Quentin en Yvelines, France), p21, Mcl-1 and cyclin B1 (Santa Cruz biotechnology, Tebu-bio, le Perray en Yvelines, France), cyclin D1 (NeoMarkers, Thermo Fisher Scientific, Illkirch, France) or HSC70 (Abcam, Paris, France) as a loading control. Primary antibodies were detected with a horseradish peroxidase-conjugated donkey anti-rabbit IgGs (GE Healthcare, Orsay, France). Blots were revealed using an Enhanced Chemiluminescence detection kit (GE Healthcare) and analyzed with the Chemcapt™ software.

### Immunofluorescence analysis

*In situ *analyses of structural proteins were performed as previously reported [15]. Antibodies sources were as follows: laminins 332 and 111 (gifts of Dr Patricia Rousselle, CNRS, Lyon), E-Cadherin (Zymed, USA), CD34 (Becton-Dickinson, USA), EGFR/c-erbB-2 (Zymed, USA), MMP7 (Chemicon International Inc. USA), collagen IV (Chemicon International Inc. USA).

Fixed frozen 7 mm tissue sections were rehydrated with 10% Fetal Calf Serum/PBS for 10 min. After several PBS washes, sections were incubated for 60 min. with the specific antibodies, washed in PBS and incubated for 30 min. with TRITC- or FITC-conjugated secondary antibodies. After two PBS washes, sections were mounted with fluorescent mounting medium (DAKO) and analyzed with a Nikon Eclips 80i fluorescence microscope. Slides were analyzed following randomization. The labelling analysis was performed blindly, and the tissue sections (serial sections) were exposed blindly to the antibodies and read by two independent observers with at least 20 microscopic fields per condition.

### RNA extraction and RT-PCR analysis

Total RNA was isolated using Trizol (Invitrogen, Cergy-Pontoise, France) and the RNA samples were used for the first-strand cDNA synthesis with the High Capacity cDNA Reverse Transcription kit and random hexamer primers (Applied biosystems). Quantitative real-time RT-PCR was performed using the Power SYBR Green Kit (Applied biosystems) according to the manufacturer's instructions. mRNA levels were analyzed in duplicate and normalized to GAPDH mRNA as an internal control. The primer sequences and reaction conditions will be provided upon request.

## Results and discussion

### 1-Statin-resistant cells grow more slowly in *Nude *mice

**Figure 1:**
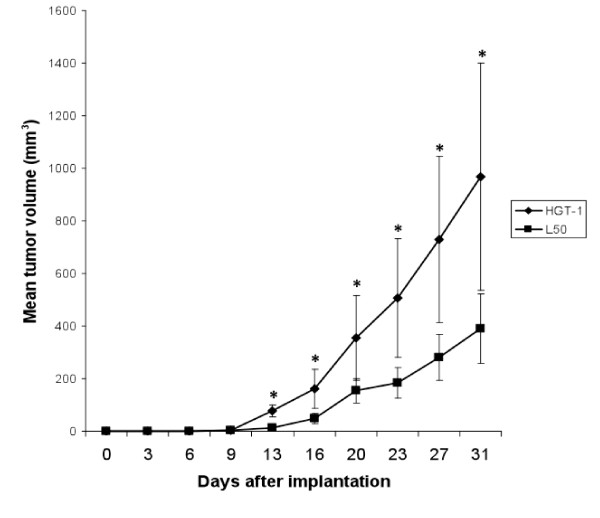
**Tumour induction in *Nude *mice**. HGT-1 or L50 cells were injected (subcutaneously) to *Nude *mice and tumour volumes were recorded as a function of time post-implantation (see Methods). Values are means ± S.D. (*n *= 10). * Statistical significance was set at the level of 5% (Student's *t *test).

We have previously shown that L50 cells had a slower growth rate *in vitro *than HGT-1 cells [11]. To determine if this was also the case *in vivo*, we injected *Nude *mice with either cell population and analysed tumour volumes up to 31 days post-implantation. As can be seen from Figure 1, HGT-1 tumours were readily detected after 13 days, whereas it required several more days for L50 tumours to become detectable. The slope of the growth curve was lower all along for L50 cells. At the end of the growth period, the mean tumour volume was 2.5 times higher for HGT-1 than for L50, with an average doubling time of 21.7 ± 5.12 days for HGT-1 cells and 25.30 ± 9.38 days for L50 cells. This difference in growth rates was statistically significant (*P*<0.05). These results demonstrate that statin-adapted cells had a slower growth rate *in vivo*, as they did *in vitro*. Nevertheless, the ability to form tumours was not impaired in L50 cells since as many mice (10 animals per group) developed tumours for both cell populations.

Expression of lipid metabolism and transport genes was reduced in L50 tumours, except for SREBP-2 that was expressed at similar levels in both tumour types (Table 1).

**Table 1:** RT-PCR analysis of marker gene expression in L50 tumour samples compared to HGT-1 tumours

Lipids synthesis	HMG-CoA reductase	0.7 ± 0.1*
	
	SREBP-1	0.6 ± 0.2*
	
	SREBP-2	n.c.
	
	LDL receptor	0.3 ± 0.06***
	
	FAS	0.6 ± 0.1**
Cell proliferation	Cyclin D1	0.7 ± 0.2***
	
	Cyclin B1	0.66 ± 0.3*
	
	P21	n.c.
	
	EGFR	n.c.

Apoptosis	Caspase 2	n.c.
	
	Caspase 3	1.5 ± 0.4**
	
	Caspase 6	1.3 ± 0.2**
	
	Caspase 7	3.9 ± 0.6***
	
	Caspase 9	n.c.
	
	Bax	1.3 ± 0.3*
	
	Bcl2	1.6 ± 0.4**
	
	Mcl-1	n.c.

Real Time RT-PCR was performed with mRNA samples extracted from 10 tumours from independent mice. The fold variation was determined with the ΔΔCt method [16]. HGT-1 mRNA levels were set to 1. The mRNA levels in L50 tumours were expressed relative to those in HGT-1 tumours. * Statistical difference (*p *< 0.05); ** Statistical difference (*p *< 0.01); *** Statistical difference (*p *< 0.001) (Student's *t *test) n.c. no change

### 2-Marker expression in HGT-1 and L50 tumours

**Figure 2:**
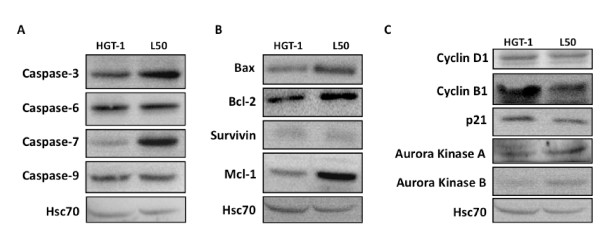
**Western blot analysis in HGT-1 and L50 tumours in *Nude *mice**. Marker protein expression was analysed by western blot in tumours from *Nude *mice, 31 days after implantation. Protein samples were prepared from pools of 6-8 mice per group. Protein levels were standardized to those of HSC-70. The data are from one experiment representative of at least three independent experiments with similar results.

To look for potential differences in marker proteins between HGT-1 and L50 tumours, we conducted a western blotting analysis. As shown in Figure 2a, pro-caspase-3 and pro-caspase-7 protein levels were higher in L50 tumours, albeit only slightly for pro-caspase 3, as had been seen in L50 cells in culture [11]. Similarly, a 3.9-fold and 1.5-fold over-expression of caspase-7 and caspase-3 mRNA levels, respectively, were also observed (Table 1). Pro-caspase-6 and procaspase-9 proteins were roughly unchanged (Figure 2a). Caspase-9 mRNA level showed no change, and caspase 6 mRNA was only marginally increased (Table 1). Both Bax and Bcl-2 protein levels were slightly raised in L50 tumours (Figure 2b), whereas Bcl-2 expression was really difficult to detect in HGT-1 and L50 cells in culture, suggesting that the reactivation of this gene was essential to allow both types of cells to develop into tumours. Bax and Bcl-2 mRNA were raised in L50 tumours (Table 1). Interestingly, increased expression of these two proteins has been shown in human gastric cancer samples [17]. In addition, an over-expression of Bcl-2 has been detected in low grade and early stage of gastric carcinomas [18].

The anti-apoptotic form of Mcl-1 was strongly raised in L50 tumours. Because L50 cells grown *in vitro *also showed higher levels of the Mcl-1 protein (data not shown), this can be interpreted as an anti-apoptotic adaptive response to growth in the continuous presence of statins during the *in vitro *selection procedure, a trait that was maintained *in vivo*. Although a high level of Mcl-1 expression was associated with a poor prognosis for many cancer types [19], its over-expression in L50 tumours might not be relevant with respect to growth potential, which clearly is a distinct endpoint from the survival of individuals who have gone through rounds of anticancer therapies. Mcl-1 mRNA levels were not raised in L50 cells (Table 1), indicating a possible stabilization event for the protein. Survivin levels were very low and comparable between both tumour types.

We next looked at the expression of proteins involved in the control of cell division. As can be seen in Figure 2c, the levels of cyclin D1, p21 and aurora kinases A & B proteins were not different between HGT-1 and L50 tumours. Strikingly, however, L50 tumours showed a strong decrease in the level of cyclin B1, as compared to HGT-1 tumours. In fact, over-expression of cyclin B1 has been positively correlated to grade, higher proliferative index, lymph node metastasis development, and invasiveness in breast, prostate and thyroid malignant lesions [20-22]. Consequently, the reduction of cyclin B1 in L50 cells might have conferred the cells a restricted proliferation potential. Cyclin B1 mRNA level was also decreased (Table 1).

Taken together, these results indicate that the slower growth of L50 tumours, as compared to HGT-1 tumours, may be attributed to a reduced activity of cell division, possibly under the control of cyclin B1.

### 3-In situ marker analysis

To analyse the relationship between tumour growth and the tumour environment, we next looked at the expression of extracellular matrix proteins, cellular junctions and neoangiogenesis markers by immunohistochemistry on serial sections prepared from HGT-1 and L50 tumours.

**Figure 3:**
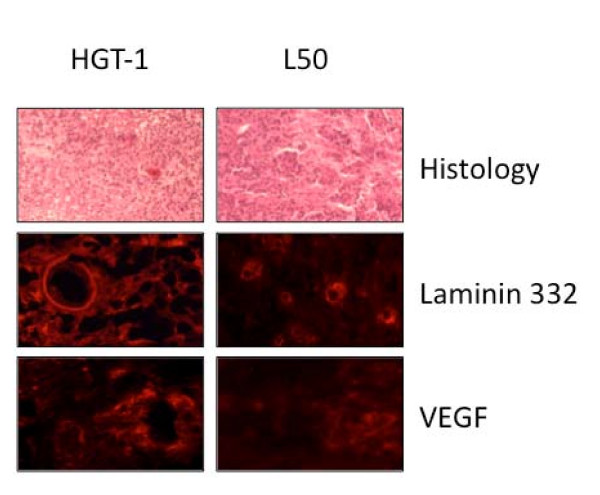
**Histology of HGT-1 and L50 tumours**. Upper panel: Representative section of HGT-1 and L50 tumours stained with hematoxylin-eosin (Original magnification × 200). Middle and lower panels: Immunofluorescence of HGT-1 and L50 tumours. Laminin 332 appeared more extensively expressed in HGT-1 tumours than in L50 tumours, namely in extracellular conjunctive spaces and also in some conjunctive cells. VEGF was well expressed in inter-tumoral spaces at the fiber and cell level. Its expression appeared stronger in HGT-1 than in L50 tumours (Original magnification × 400)

Morphology analysis showed that HGT-1 tumours were less differentiated than L50 cells, which also appeared more rounded. In addition, L50 tumours showed a lobular structure, which was not observed in HGT-1 tumours (Figure 3).

The extracellular matrix protein laminin 332 was less expressed in L50 than in HGT-1 tumours, whereas the reverse was true for collagen IV (Table 2). All the tumours expressed Laminin 332, but its localization was variable between tumour types: it was localized between the vessels of HGT-1 tumours and in the cell cytoplasm and intercellular spaces in L50 tumours, but at rather low levels. Laminin 111 was strongly expressed, and at similar levels in both tumours. Nevertheless, since laminin 111 is never produced by epithelial cells, we may speculate that the observed laminin 111 staining could have been contributed by other cells. Collagen IV staining was more marked in L50 than in HGT-1 tumours (Table 2), namely at the level of blood vessels. In addition, a few cells, probably fibroblasts, expressed collagen IV in the conjunctive tissue space.

**Table 2:** Semi-quantitation of protein levels from the immunofluorescence analysis

	HGT1	L50
Laminin 332	++	+

Laminin 111	++	++

Collagen IV	+	++

E-Cadherin	+	+

CD34	++	+

VEGF	++	+/-

c-erbB-2	+	++

MMP7	+	++

A semi-quantitative analysis of fluorescence signals in tumour cells was tabulated. Each analysis was based on the observation of at least 20 microscopy fields.

E-cadherin, an epithelial and endothelial cell junction marker, which abnormal expression has been implicated in hereditary forms of gastric cancer [23], did not display obvious differences between HGT-1 and L50 tumours, but was well expressed by the host vessels.

The neoangiogenesis CD34 marker appeared more expressed by HGT-1 tumours (Table 2), which supports the notion that angiogenesis was more active in HGT-1 than L50 tumours and, presumably, could have contributed to the higher growth rate of HGT-1 tumours when compared with L50 tumours. In addition, VEGF was strongly expressed in HGT-1 tumours but was barely detectable in L50 tumours (Figure 3 & Table 2).

c-erbB-2, a receptor tyrosine kinase member of the EGFR family, involved in growth control, cell adhesion, migration and differentiation, was obviously expressed by stromal cells between tumour islets. In addition, L50 tumours appeared to express more c-erbB-2 than HGT-1 tumours (Table 2).

Matrilysin 1 (MMP7), which is generally strongly involved in the proteolytic process of the stroma, appeared more expressed by L50 than by HGT-1 tumours at the cell level. This may seem contradictory, as the consequence of such over-expression should be increased metastatic potential for L50 cells. Nevertheless, we obtained no evidence for metastases in the course of this study. It may be possible that the threshold level for tumour cells to evade from the tumour and form metastases was not attained.

To summarize, when compared to HGT-1 tumours, L50 tumours displayed over-expression of collagen IV, c-erbB-2 and MMP7, and reduced laminin 332, CD34 and VEGF levels. These results indicate that the restriction in L50 tumour growth may be largely explained by a more differentiated phenotype, and a reduced ability to undergo tumour neo-angiogenesis. Hence, it can be hypothesized that gastric cancers developed in individuals with a long history of statin treatment, might be more responsive to targeted chemotherapy including trastuzumab or lapatinib, c-erbB-2 inhibitors, which anticancer potentials are currently being evaluated in several clinical trials [24]. From an experimental perspective, we plan to develop a large-scale proteomic approach with the aim of identifying additional markers that could also participate in the reduction of growth potential for statin resistant cells *in vivo*. This type of approach has been recently developed [25] with breast cancer cells *in vitro *following treatment with lovastatin, but we are not aware of any study that would have analyzed the proteome of cells selected for their resistance to statins. Interestingly, no overlap between our results and those from this analysis was observed. Although this may in part be due to the difference in cell types (breast *vs *stomach), we surmise that the situations - short-term treatment with lovastatin or selection of lovastatin-resistant cells - may not be comparable at all. Future experiments should shed new light on this question.

## Conclusion

This study showed that adaptation to the continuous presence of statins *in vitro *led to a growth phenotype that was inherited and largely maintained in tumours formed in *Nude *mice. Hence, growth was reduced in statin-adapted tumour cells while showing a higher degree of differentiation and a restricted ability to undergo neo-angiogenesis. The elevation in the levels of pro-caspase-3, pro-caspase-7 and Bax in L50 tumours suggested that they might be more sensitive to anticancer treatments than HGT-1 tumours, since they expressed a higher pool of pro-apoptotic molecules, presumably prone to drug-induced activation. Moreover, that the level of cyclin B1 was lower in L50 tumours suggests that they might be less aggressive and, possibly, more responsive to chemotherapy, a hypothesis that we plan to test in the future. As a model with these HGT-1 and L50 cells, it can be proposed that cancer cells that would appear in individuals undergoing statin therapy would be less likely to develop into tumours. Such effects could account, in part, for the lower incidence of several types of cancers in humans treated by statins for several years, as has been suggested in several retrospective studies [3,4]. Future trials could help evaluate this hypothesis through the analysis of markers in tumours from naïve or statin-treated subjects.

## Competing interests

The authors declare that they have no competing interests.

## Authors' contributions

JF, BS and CLJC performed the cell culture, western blotting and RNA analyses; LR and DG performed the immunohistochemistry analyses; VH and PA performed the mouse analyses; CLJC and LC designed the experiments, analyzed the data and wrote the article.

## Pre-publication history

The pre-publication history for this paper can be accessed here:

http://www.biomedcentral.com/1471-2407/11/491/prepub
